# Printing Multistrain Bacterial Patterns with a Piezoelectric Inkjet Printer

**DOI:** 10.1371/journal.pone.0000663

**Published:** 2007-07-25

**Authors:** Jack Merrin, Stanislas Leibler, John S. Chuang

**Affiliations:** Laboratory of Living Matter and Center for Physics and Biology, The Rockefeller University, New York, New York, United States of America; Center for Genomic Regulation, Spain

## Abstract

Many studies involving interacting microorganisms would benefit from simple devices able to deposit cells in precisely defined patterns. We describe an inexpensive bacterial piezoelectric inkjet printer (adapted from the design of the POSaM oligonucleotide microarrayer) that can be used to “print out” different strains of bacteria or chemicals in small droplets onto a flat surface at high resolution. The capabilities of this device are demonstrated by printing ordered arrays comprising two bacterial strains labeled with different fluorescent proteins. We also characterized several properties of this piezoelectric printer, such as the droplet volume (of the order of tens of pl), the distribution of number of cells in each droplet, and the dependence of droplet volume on printing frequency. We established the limits of the printing resolution, and determined that the printed viability of *Escherichia coli* exceeded 98.5%.

## Introduction

Besides commercial printing of ink on paper, there are many other useful applications of inkjet technology. In the electronics industry, inkjet printing finds use in printing electronic circuits using conductive polymer “inks”[1], in maskless photolithography[2], or in creating low cost and flexible polymer light-emitting diode (PLED) displays by printing electroluminescent conductive polymers[3]. A number of biological applications have been developed. One example, POSaM[4] (Piezoelectric Oligonucleotide Synthesizer and Microarrayer), achieves *de novo* synthesis of oligonucleotide microarrays by using an inkjet printhead to deposit phosphoramidite precursors and a tetrazole activator at precise locations on glass slides. Other examples include printing of bacterial colonies[5], adhesion substrates for patterning neuronal cells in culture[6], protein arrays[7], and patterned growth of mouse myoblast cells on surfaces coated with inkjet printed growth factors[8]. The printing of tissues or organs may be eventually possible by extending high throughput 2D methods for patterned cell attachment and cell printing[9].

There are two main classes of inkjet printers, thermal and piezoelectric. In thermal inkjets, a resistive heating element causes air bubbles to expand, expelling a liquid drop. In piezoelectric inkjets, voltage-induced deformation of a rectangular piezoelectric crystal squeezes ink droplets through the nozzle. POSaM employs piezoelectric inkjets because they are able to print a wider variety of solvents and because they are easier to clean.

We have adapted POSaM to create a simple piezoelectric printer for patterning bacteria onto a substrate such as a glass slide, agar plate, or nitrocellulose membrane. Our motivation for developing a bacterial inkjet printer is to enable precise control of the spatial arrangement of interacting microbial strains. For example, different strains could be patterned in lattices, grids, rows, or other geometries. Inkjet printing not only allows us to vary the spacing of such arrangements, but also allows higher inter-drop resolution than nl dispensers because of the small drop volumes (typically less than 30 pl). Automated control of printing would result in reproducible initial conditions important for the quantitative analysis of patterned growth on agar surfaces or membranes.

Previously, colony arrays of a single bacterial strain have been printed using thermal inkjets[5]. Our work establishes that printing of bacteria can also be done using piezoelectric inkjets. In particular, we demonstrate printing of multiple cell types in ordered arrays. We also describe various characteristics of this printer including droplet properties and cell viability.

## Methods

### Printer setup

**Figure 1:**
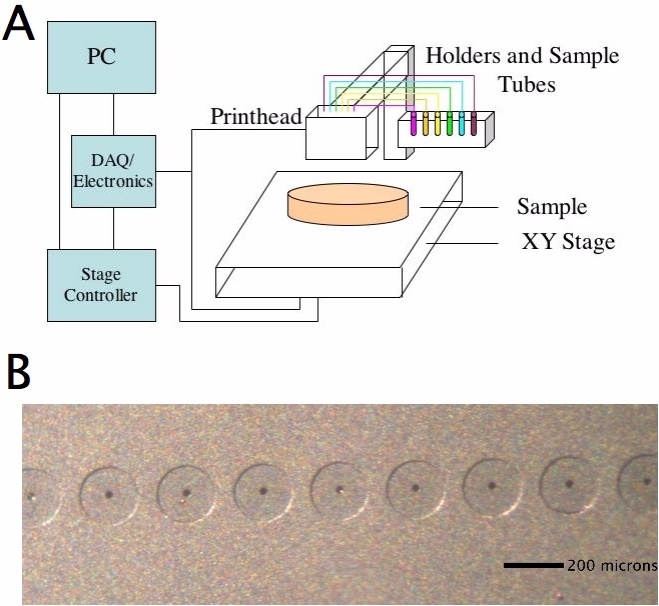
System overview. (A) Schematic view of the printer set-up. (B) Microscopic image of the printhead. The inkjet nozzles (small black centers) are spaced 282 microns apart and have a diameter of 36.0±2.6 microns.

Our printing system, based on the POSaM design[4], is assembled from an Epson F057020 printhead, a motorized stage, a rack of bottle holders for inks, a PC, and control electronics as shown in Figure 1A. The printhead contains six parallel linear banks of 32 nozzles each, with each bank connected to a different ink source (Figure 1B). An aluminum platform stage was machined and agar plates were secured onto the stage with modeling clay. The platform stage was motorized by attaching it to a XY-table with 8.5x9.5 inches of travel, a step size of 2.5 µm, and 10 µm back and forth repeatability (Velmex MA2512K1J-S2.5 and MB2512K1J-S2.5 with a VXM2 stepper motor controller).

### Electronics and software

The electronics consists of a multifunction data acquisition (DAQ) board and a circuit board. Digital waveforms generated by a DAQ board, AT-DIO 32HS (National Instruments), were converted to trapezoidal pulses by the circuit board electronics with a maximum height 30V. These waveforms drive the piezoelectric crystals inside the printhead to produce drops. The circuit board is a simplified version of the electronics of the POSaM project which implements waveform generation, droplet detection, and solenoid array controls. The DAQ board also sends digital pulses to the motorized stage controller prompting it to move to the next coordinate in preprogrammed raster patterns. The cable connecting the printhead to the electronics was obtained by scavenging an Epson Stylus 700 printer. Software which controls the electronics was written in Visual Basic 6.0 and contains custom routines for printing patterns by directing stage motion, enabling droplet formation from arbitrary nozzles, and generating arbitrary voltage waveforms. The POSaM software was used as a starting point. It is freely available at www.bioinformatics.org/pogo/.

### Fluid loading and tubing connections

The solution to be printed (e.g. cells, beads, or ink for test runs) is loaded into a 5 ml septa-capped vial (Fisher Scientific). A rack secured close to the printhead can hold six of these vials. A 0.032” outer diameter, 2” long stub needle (Small Parts Inc.) pierces the septum down to the bottom of the vial and is connected to 0.030” inner diameter silicone tubing (Tygon, Cole Parmer), ending with a tubing adapter (3/16” outer diameter, 1/16” wall; Pharmed) to the printhead inlets. A syringe and two 25 gauge needles that pierce the septa are used to prime the nozzles. One needle attached to the syringe applies pressure while the second needle is temporary sealed. During priming, air pressure generated within the syringe causes large droplets or jets to be expelled through the printhead nozzles. Removing the temporary seal releases the pressure to equalize with atmospheric and then nozzle surfaces are wiped clean with a lintless towel. A cleaning procedure is used between printing sessions which consists of flushing nozzles and the tubing with acetone and then air drying.

### Selection of functioning nozzles

After repeated use, it is difficult to ensure that all the nozzles will fire after priming the printhead so they must be individually tested. Nozzles usually fail permanently if they are clogged, or temporarily if either an air bubble enters the nozzle or if liquid pools underneath the nozzle. Such hanging droplets may be due to pressure differences between the vial and the nozzle. After priming, it is important to inspect the inkjet head to ensure that hanging drops are not forming, since these prevent drops from falling onto the substrate. Individually functioning nozzles were identified using one of two droplet detection schemes. The first method used was to print a thousand droplets from individual nozzles onto parafilm or a piece of paper. A visible droplet or a colored spot can then identify functional nozzles. The second method we used to identify functional nozzles was to print a pattern of columns with colored solutions onto a piece of paper. Missing columns are associated with malfunctioning nozzles.

### Bacterial strains

The strains printed are derived from wild type *E. coli* strain MG1655. Fluorescent cells were constructed by transforming plasmids pZS*2R-CFP, pZS*2R-GFP, or pZS*2R-Venus. These plasmids are based on the low-copy pZS* vector series[10] and express the fluorescent protein from a strong constitutive promoter. In some experiments, where cell adhesion inside the printhead and tubing was a concern, non-sticky cells from strain NS2 were used. NS2 is an MG1655 derivative (constructed in our laboratory by Doeke Hekstra and Sri Ram) containing knockouts[11], [12] of the *fimA* gene which encodes the main structural protein of fimbriae allowing bacteria to stick to surfaces and of the *flu* gene which encodes the Ag43 auto-aggregation protein.

### Estimation of drop volume

To determine how well the printer could be calibrated, we measured drop volumes by three different methods. The first method consisted of direct counting under the microscope of the number of beads or cells inside droplets printed from a solution of known concentration. The second method consisted of weighing 500,000 drops and then dividing the mean weight of one droplet by the density of water. The third method was to print a fluorescent solution, e.g. of fluorescein droplets, fluorescent cells, or fluorescent beads, and compare the fluorescence of 100,000 printed droplets (∼3 µl) to a standard calibration curve of fluorescence versus volume. Fluorescence measurements were performed in multiwell plates using a Wallac Victor2 fluorimeter (PerkinElmer).

### Printing of multiple strains

A matrix encoding the locations where each strain is to be printed was created by our printing software. The motorized stage was programmed to move in a raster pattern, and at each point, a nozzle from the first bank was activated if strain 1 was to be printed at the current location. The stage was then homed and shifted by an offset (accounting for the distance between different banks) and the stage was moved again, this time firing the nozzle from the second bank to print strain 2 when appropriate.

Slight variations in nozzle shapes may lead to small angular deviations of the droplet trajectory. For example, at a substrate distance of 5 mm, a 1° angular deviation would result in a (5 mm)(tan1°) = 43.6 µm horizontal displacement. Therefore, the offset was fine-tuned when the distance from the nozzle to the substrate caused significant droplet deflection. For immediate visual feedback of printing accuracy, we typically added a colored dye to the bacterial solutions. Ink for normal desktop printing on paper, Generations Micro-Bright Ink (light-cyan or light magenta; from inkjetmall.com), was originally used because the dye did not spread after printing, but this ink was found to have negative effects on cell viability. We determined that these three inks have a negligible effect on cell viability at the following concentrations: 2.5 mg/ml Allura Red, 2.0 mg/ml Bromophenol Blue, and 0.5 mg/ml Xylene Cyanol.

### Image Acquisition and Analysis

Phase contrast and fluorescence images of cells in individual droplets were acquired with a Retiga EXi CCD camera (QImaging) attached to a Zeiss Axiovert 200M microscope. Images were analyzed for cell counts and cell positions using ImagePro (Media Cybernetics). Images of fluorescent colonies patterned on agar plates were acquired using a Typhoon 9400 fluorescent scanner (GE Amersham Biosciences). The resolution of an array pattern was determined by extracting colony locations using ImagePro. The position data were the analyzed in Matlab (Mathworks, Inc.) as follows. For each row, end colonies were assumed to be in position. The absolute horizontal deviations of all the colonies between the end colonies from an even spacing were determined and averaged over several rows. The y deviation was assumed to be the same as the x deviation.

## Results and Discussion

### System Overview

The piezoelectric printer is outlined in block diagram form in Figure 1A. Solutions containing cells to be printed sit in a sample holder. Samples are connected to the printhead inlets via tubing. The inkjet printhead is fixed in place. A substrate (e.g. agar plate, membrane, or glass slide) is fastened to a motorized stage, such that movement of the stage allows the inkjet to print at different locations on the substrate. A computer running custom software coordinates stage movements by relaying messages to the stage controller and also triggers inkjet nozzle actuations by sending appropriate voltage waveforms to the printhead via a DAQ card and an electronic circuit board. Our circuit board design is a simplified version of the POSaM electronics and includes only functions related to inkjet actuation. With the printer constructed, we proceeded to test the viability of printed cells and to characterize drop properties.

### Viability of printed cells

**Figure 2:**
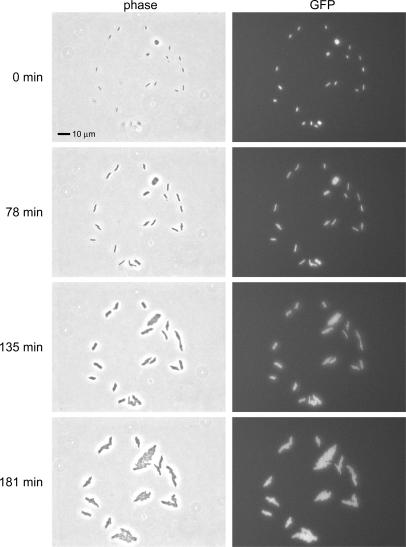
Viability of printed *E. coli*. A timed progression of phase contrast microscopy images shows cells printed in a single droplet growing over 3 hours at 30°C. The corresponding fluorescence images show that all the cells in this droplet are viable. The overall viability was greater than 98.5% (see text).

Large shear forces are present when droplets exit the printer which may harm the bacteria. In order to test the viability of piezoelectrically printed fluorescent *E. coli*, single droplets of a fresh overnight culture of MG1655 + pZS*2R-GFP were printed onto an agar plate. Using phase contrast and fluorescence microscopy, 273 cells were tracked for three hours during growth at 30°C. The fluorescence allowed us to distinguish between live cells and lysed cells or dust, but phase contrast was sufficient to identify all the growing cells. Figure 2 shows one field of view of a subset of the cells tracked. Four events of the total dataset, identified as possible dead or damaged cells, either did not grow, did not divide, or were not fluorescent indicating lysis. We could not determine whether these four events were a consequence of the printing process or whether they were already present in the overnight culture. We concluded that at least 98.5% of the bacteria are viable after being printed through the printer. In a different experiment, Xu *et. al* determined that over 92% of mammalian cells were viable after being printed through a thermal inkjet printhead[13].

### Drop volume estimation and cell number statistics in individual droplets

The droplet volume generated by a piezoelectric inkjet depends on factors such as the nozzle geometry, the shape of the voltage waveform applied to the piezo, actuation waveform frequency, solvent viscosity, and solvent surface tension. The average concentration of cells in printed droplets *c*, the average number of cells per droplet *n̅*, and the droplet volume *V_d_* are related by
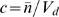



The first method we used to estimate drop volume is based on a simple assumption that the number of cells counted per droplet is expected to produce a Poisson distribution, where the probability of finding *n* cells per drop with an average of *n̅* is
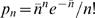



**Figure 3:**
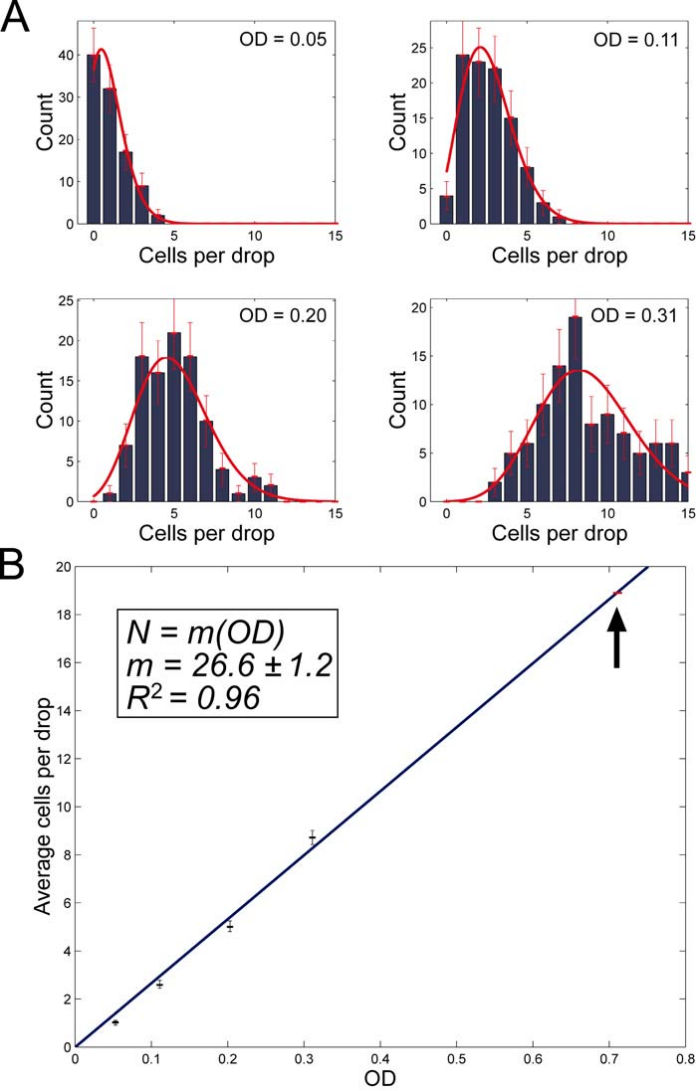
Poisson distribution of cell number per droplet and drop volume determination. (A) The distribution of cells in the individual droplets are depicted for four different densities. Red curves are theoretical Poisson distributions matched to the mean number of cells per droplet. The measured means were used to determine the drop volume (see text). (B) The average number of cells per droplet N, versus optical density, OD_600_. The curve fit is a linear least squares fit to the equation in the box. *R^2^* is the Pearson product-moment correlation coefficient which is a measure of linearity of the data. The calibration with flow cytometry was done at OD_600_ = 0.71 (black arrow).

To test this assumption, samples containing fluorescent GFP-expressing cells at four different optical densities, were printed onto glass slides. The stage moved after each drop, so the printing rate was 1 drop every 0.1 seconds. For each sample, the number of cells per droplet was measured by microscopy for 100 different droplets (Figure 3A). The expected Poisson distributions (red curves) are plotted using the means of the four measured distributions in Figure 3A. The Poisson distribution indicates that cells are sorted randomly in an independent fashion into individual droplets.

From the above equation, we obtained the mean droplet volume *V_d_* by dividing the measured mean cells per droplet by the known cell concentration. The correspondence between cell density and optical density was calibrated by counting bacteria via flow cytometry. At an optical density of 0.71 the cell density measured on a flow cytometer was 1.07±0.11×10^9 ^cells/ml. Below this cell density, OD_600_ was found to be linear with cell density by successive two-fold dilutions (Figure 3B). For single droplets, printed every 0.1 seconds, the mean drop volume is then estimated as 15.3±3.4 pl.

### Positional distribution of cells within droplets

**Figure 4:**
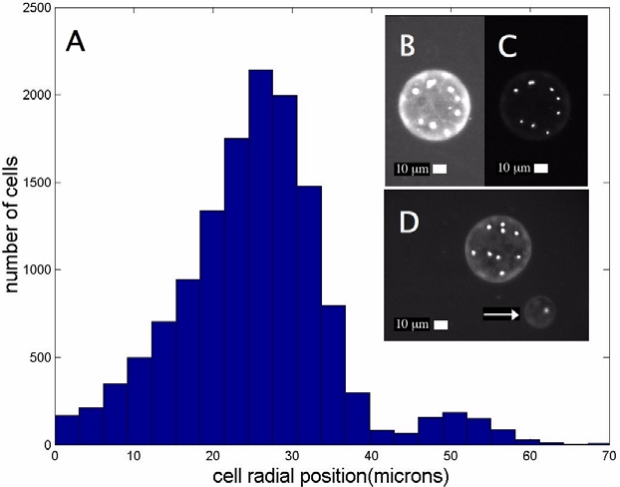
Positional distribution of cells within printed droplets. Insets (B), (C), & (D): Fluorescent images of droplets containing fluorescein and fluorescent bacteria, printed onto glass. (D) A typical droplet and with it a nearby satellite droplet (arrow). The satellite droplets give rise to a secondary peak in the radial distribution (A). The long photo exposure in (B) is used to clearly determine the outline of the droplet relative to the position of cells as seen at a short exposure (C).

The images obtained to determine the distribution of cell numbers also contain information about the positional distribution of cells and wetting of droplets. The number of bacteria and their positions were determined relative to the center of the droplet by image analysis. The distribution of cell locations in fluorescein droplets on glass is shown in Figure 4A. Cells were visualized with GFP, and the droplet outlines were visualized by adding a fluorescein to the solution at a concentration of 1 mg/ml (Figure 4BCD). The GFP signal is an order of magnitude stronger than that of the fluorescein. If one assumes the droplet spreads evenly as a cylindrical section, and that cells distribute uniformly, then the radial distribution would be *u(r)dr ∝ rdr*. This form predicts a linear increase in the frequency of cells at a radius *r* from the origin up to the average radius of a droplet. The observed distribution may indicate a slight preference of cells to be pushed towards the perimeter. Smaller satellite droplets (Figure 4D) can occur if the nozzle becomes locked into a particular state. These satellite droplets gave rise to a second peak in the radial distribution in our dataset. One out of five of our nozzles had produced satellite droplets in this particular experiment and only 6.8% of the total cells fell within a satellite droplet. Even if satellite droplets occur within a distance of two to three droplet spreading radii, a reasonable pattern resolution can still be obtained for the larger picture. To print pristine bacterial patterns the quality of the nozzles must be first determined.

### Printed arrays of multiple strains

**Figure 5:**
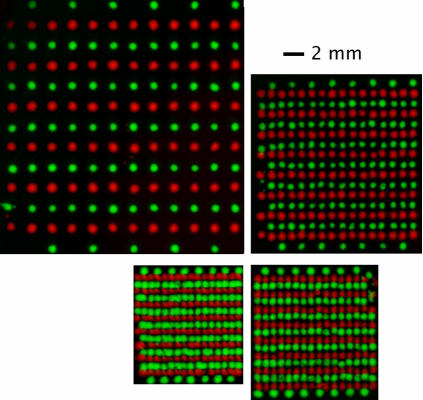
Colony array printing of two types of fluorescently labeled cells. CFP-labeled cells in (pseudo color) red and Venus-labeled cells in (pseudo color) green. The spacing between adjacent colonies is 2 mm, 1 mm, 0.5 mm, and 0.35 mm. The 2 mm scale bar is common to all 4 pictures.

Array patterns with two color cell types, (MG1655 cells containing pZS*2R-CFP and pZS*2R-Venus plasmids) were printed with 10 drops per location at an approximate density of 10 cells per droplet. A series of lined patterns were printed to determine the smallest spatial resolution possible. Figure 5 shows sample plates of the two cell types printed at four different spacings: 2 mm, 1 mm, 500 µm or 350 µm and grown into colonies for 10 hours at 30°C. When the diameter of colonies grew to 375±165 µm, the error in colony placement (defined in Methods) was 31.9±22.5 µm. The printing resolution we observed, at ten drops per location, was about 350 microns, but we should be able to achieve a higher resolution by printing colonies from single droplets. The error in placement is smaller than a droplet spreading diameter indicating the center is mostly determined by how cells coalesce and not the ejection angle from the printer or the movement of the motorized stage.

**Figure 6:**
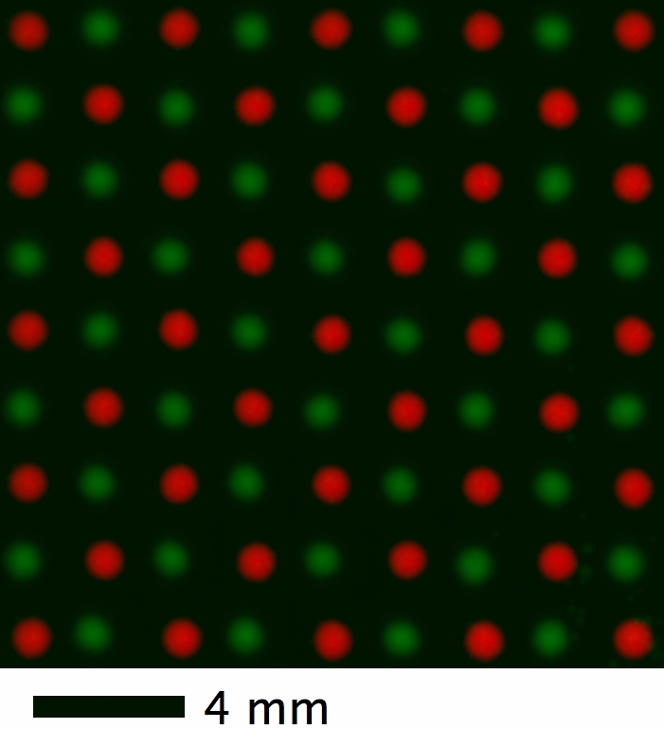
Checkerboard pattern printed with two strains. CFP-labeled and YFP-labeled cells are shown respectively in pseudo color red and green.

We also demonstrated the possibility of printing more complex patterns, such as a checkerboard shown in Figure 6, indicating that the placement of eventual colonies is accurate with two cell types for programmed patterns. The biology of how adjacent colonies grow, spread, and interact is beyond the scope of this paper. We observed it takes about 1–2 days at 30°C for colonies to merge symmetrically into rectangular colonies when they are printed at close distances less than 2 mm (data not shown). It will be of interest to study how ecological interactions (cooperation, competition, and fitness differences) affect the shape boundary.

### High frequency printing

The two strain patterns in Figure 4 were printed by a relative low frequency actuation process. The stage moves to a new point, the inkjet fires after some delay, and the stage moves to the next point, and so on. In principle, one may want to print cells more rapidly, so it was of interest to examine drop properties at higher firing frequency. In reference [14], theoretical calculations and empirical results show how the volume of droplets can change as a function of printing frequency by a factor of two. At sufficiently high jetting frequencies, residual vibrations in the printhead may not have enough time to fully dampen, resulting in changes in drop volume and reproducibility.

To investigate drop properties when printed at high frequency, we printed fluorescent *E. coli* or fluorescein drops at 10 kHz. We obtained the mean drop volume by printing 500,000 drops and either weighing the printed volume or measuring the fluorescence of the printed volume and using the calibration curve. For such high speed printing we found the drop volume was 30.6±8.7 pl by direct weighting (116 weighing measurements), and 30.2±2.7 pl in an experiment of fluorescein droplet fluorimetry.

For low speed printing, on the other hand (Figure 3), the measured drop volume was 15.3±3.4 pl as a result of analyzing the counting statistics of 500 droplets printed at different cell densities. We conclude that for applications in which the printed drop volume is critical, it is necessary to calibrate the volumes at the desired printing frequency. However for most applications, such as depositing different patterns of bacterial strains, a change in drop volume by a factor of two is tolerable.

### Possible Improvements and Future Applications

The current design can be improved in several ways. The motorized stage was the limiting factor to printing speed due to specifics of the controller. Alternative stages could be used or built at even lower cost. A motorized z-axis would also be useful for precisely raising and lowering the inkjet printhead assembly so that the height of the nozzles relative to the substrate could be more reproducibly controlled. This could be of some concern because at large enough distances (>5mm), droplet deflection may become significant, thereby degrading printing resolution. In addition, the relatively expensive DAQ card could be replaced with a simple, high-speed microcontroller such as the Atmel AVR or Microchip PIC which would considerably reduce the cost of the electronics.

Our bacterial piezoelectric inkjet printer should find many applications such as synthetic biology and model “ecological” studies involving microbes. For example, multicellular systems of engineered bacterial strains can display interesting spatial patterns of gene expression[15]. The printer could be used to alter the initial spatial arrangement of the strains to explore its effect on pattern formation. In addition, ecological interactions between communicating, cooperating, or competing strains could be systematically investigated by printing the strains in a variety patterns. Potentially, biofilms of predetermined spatial structure consisting of multiple species could also be printed. A natural extension to our methods explored here includes direct printing of antibiotics or other active molecules to different surface locations to observe inhibition or excitation of printed biological landscapes. Thus, by the arraying of multiple cell types many new possibilities in microbiology experiments become available at relatively low cost.
